# Antioxidant defense capacity of ovarian tissue after vitrification in a metal closed system

**DOI:** 10.5935/1518-0557.20180044

**Published:** 2018

**Authors:** Eloísa T. Massignam, Maitê Ferreira, Eduardo Sanguinet, Ágata Dupont, Fábio Klamt, Nilo Frantz, Adriana Bos-Mikich

**Affiliations:** 1 Department of Biochemistry, ICBS, Federal University of Rio Grande do Sul, Porto Alegre, RS, Brazil; 2 Department of Morphological Sciences, ICBS, Federal University of Rio Grande do Sul, Porto Alegre, RS, Brazil; 3 Nilo Frantz Human Fertility Center, Porto Alegre, RS, Brazil

**Keywords:** Ovarian tissue, vitrification, oxidative stress

## Abstract

**Objective:**

The present study analyzed the quality of bovine ovarian tissue after vitrification in a metal closed chamber, in terms of putative changes in tissue viability (lactate dehydrogenase -LDH- release), anti-oxidant defenses, and redox parameters caused by cryopreservation.

**Methods:**

Small and large fragmented bovine ovarian tissue specimens were vitrified in a metal chamber. After rewarming, tissue samples were fixed or cultured for 48 hours. Glutathione (GSH), protein sulfhydryl content, Total Radical Trapping Antioxidant Potential (TRAP), and lactate dehydrogenase were analyzed immediately after rewarming and after tissue culture.

**Results:**

No changes in antioxidant parameters or viability of rewarmed tissue samples were found immediately or 48h after vitrification. The method of vitrification in a metal closed chamber used in this study preserved the quality of bovine ovarian tissue. Furthermore, our data showed that the size of the tissue specimens did not affect post-vitrification biochemical viability parameters.

**Conclusions:**

We believe that the vitrification methodology employed in the present study is safe and effective, and should be evaluated for use in humans.

## INTRODUCTION

Ovarian tissue cryopreservation is an option for the restoration of hormonal and reproductive function of females facing cancer. Presently, ovarian tissue cryopreservation is primarily performed using slow freezing protocols. However, comparative studies have demonstrated that vitrification might more effectively preserve ovarian structure and function (Tokieda *et al*., 2002; Keros *et al*., 2009). Most vitrification procedures employ an open system to directly expose the biological material to liquid nitrogen (Sugimoto *et al*., 2000; Tokieda *et al*., 2002; Migishima *et al*., 2003; Almodin *et al*. 2004; Chen *et al*., 2006; Keros *et al*., 2009; Isachenko *et al*., 2009; Sheikhi *et al*., 2011; Fabbri *et al.,* 2010). Considering that the consequences of cryostorage can only be confirmed over long periods of time, and since it is impossible to guarantee there will never be any cross-contamination, current recommendations support the use of systems in which there is no direct contact between the cryopreserved material and liquid nitrogen (Pomeroy *et al*., 2010). Our group has developed a metal chamber in which ovarian tissue specimens are vitrified without direct contact with liquid nitrogen (Aquino *et al.,* 2014). Histological analysis of rewarmed tissue specimens showed well-preserved stroma, primordial, and primary follicles (Marques *et al*., 2015; Aquino *et al*., 2014).

However, *ex-vivo* tissue manipulation and cryopreservation may induce oxidative stress, thus damaging cells and compromising tissue viability and function after re-transplantation. Reactive oxygen species (ROS) are unstable molecules produced during normal cell physiological processes. These molecules play important roles in cell signaling, including processes involved in reproduction (de Lamirande & Gagnon, 1995; Shkolnik *et al*., 2011; Vu *et al*., 2012; Silva *et al*., 2015). However, ROS have also been associated with adverse effects on reproduction, particularly on gametes, including impaired embryonic development and diseases in the offspring (Ruder *et al*., 2008; Xiao *et al*., 2012).

Under normal physiological conditions, cells and tissues produce and store antioxidant agents, especially glutathione (GSH) and protein sulfhydryl groups, to counteract oxidant effects and defend cells from ROS. Imbalances between ROS production and defense mechanisms protecting cells against damaging oxidative molecules result in oxidative stress. Tissue and cell manipulation may lead to excessive ROS production, which may in turn lead to cell and tissue damage and eventually death.

There is no information on whether the vitrification process compromises the antioxidant defense capacity provided by the intracellular defense mechanisms of oocytes, follicular cells, and stroma of the ovarian tissue. Although thawed graft performance after transplantation is the ultimate validation of any ovarian tissue cryopreservation method, biomarkers are needed to predict tissue viability after cryopreservation.

The aim of this study was to assess the viability of bovine ovarian tissue specimens using a metal closed system for vitrification by measuring the tissue levels of GSH and protein sulfhydryl groups, total radical trapping anti-oxidant potential, and lactate dehydrogenase production in cryopreserved specimens immediately after rewarming and after culture.

## MATERIALS AND METHODS

### Tissue manipulation and vitrification

Bovine ovaries were collected at a local abattoir and transported to the laboratory within two hours of slaughter in a sterile glass vessel containing saline solution at room temperature (RT; ~23ºC). Cortex slices were cut in two sizes: 1x1x3 mm (small fragments “S”) and 1x1x5 mm (large fragments “L”) with a scalpel. The specimens were transferred first to equilibrium solution (ES) with 7.5% ethylene glycol (EG) and DMSO and then to vitrification solution (VS) with 15% EG and DMSO, both in HTF (Irvine) medium for 25 and 15 min, respectively. The fragments were gently transferred from one solution to the next with the help of fine sterile paintbrushes to avoid tissue damage and to carry the least amount of medium in each transfer. Ten to 12 tissue specimens from different ovaries were placed in the bottom of a metal cryovial (Patent no.: BR 20 2013 019739 0); a lid was tightly fastened on the top of the vial and the system was immersed in liquid nitrogen (LN2) for storage from one week to two months (Aquino *et al.*, 2014). For each experimental replicate, four to five fresh samples from different ovaries were transferred directly to lysis buffer (0,25M sucrose, 1mM EDTA, 10mM Tris-HCl (pH7.5), 20% glycerol, 0.1% phenylmethylsulfonyl) to be used as fresh controls for biochemistry assays and then stored at -80ºC. The Institutional Ethics Committee of the Federal University of Rio Grande do Sul approved the study (permit no. 25088).

### Tissue rewarming and culture

The cryovials were pulled out of liquid nitrogen and exposed to tap water for 30 sec to allow the lids to be unfastened. Then, the bottoms of the cryovials were immersed in water at 37º C for 1 min. The rewarmed contents were gently removed from the bottom of the capsule and transferred to the first warming solution containing 1M sucrose for 1 min, followed by the second solution containing 0.5M sucrose for 3 min, and the last solution containing 0.25M sucrose for five minutes. All solutions were at room temperature. After rewarming, the fragments were transferred to lysis buffer and stored at -80º C or cultured for 48 hours in HTF medium at 38.5ºC. Fresh tissue samples from different ovaries were placed under the same culture conditions (CG/CS, controls). After culture, the specimens were transferred to lysis buffer and stored at -80ºC for further analysis. The culture media were transferred to Eppendorf tubes containing 500 µL of lysis buffer and stored at -80ºC for LDH assay.

Three experimental replicates were carried out.

### GSH, Total Radical-Trapping Antioxidant Potential (TRAP), Reduced Thiol (−SH) and LDH Levels

### Reagents and Equipment

All reagents were obtained from Sigma-Aldrich Brazil (São Paulo, Brazil), except when otherwise indicated. Spectrophotometric measurements were assayed in a 96-well microplate reader (SpectraMax i3, Molecular Devices).

### Tissue preparation

The ovarian tissue fragments were transferred to a 25 mL glass Potter-Elvehjem Homogenizer containing 500 µL of lysis buffer. The tissue specimens were struck several times with a pestle until a homogenate was obtained. The homogenate was centrifuged at 500rpm for 5 minutes and the supernatant was collected for protein, GSH, sulfhydryl, and TRAP testing.

### Total Protein Content Quantification

Bradford assays were performed to measure the protein levels of each experimental group and repeats (Zor & Selinger, 1996). All samples were diluted with lysis solution.

These values were used to correct GSH, sulfhydryl, and TRAP test results.

### Reduced Glutathione Concentration Assay

GSH concentrations were measured according to Browne & Armstrong (1998) with minor modifications. In summary, the samples (1 µg protein/µL) were first deproteinized with meta-phosphoric acid, centrifuged at 7000g for 10 min, and immediately used for GSH quantification. 185 µL of 100 mM sodium phosphate buffer (pH 8.0) containing 5 mM ethylenediaminetetraacetic acid and 15 µL of o-phthaldialdehyde (1 mg/mL) were added to 30 µL of previously deproteinized supernatant. The mixture was incubated at room temperature in a dark room for 15 min. Fluorescence was measured using excitation and emission wavelengths of 350 and 420 nm, respectively. The final quantity of glutathione was calculated using a GSH standard curve (Browne & Armstrong, 1998). Values were corrected for the protein levels determined by the Bradford assay.

### Reduced Thiol (-SH) Levels Assay

Sulfhydryl group (−SH) levels were determined by measuring absorbance of DTNB at 412 nm. DTNB 10 mM (5,5’-dithionitrobis 2-nitrobenzoic acid) was added to the samples and sulfhydryl levels were determined by reacting samples with 5-thio-2-nitrobenzoic acid (Nbs). Results were expressed in nanomoles of sulfhydryl per µg of protein (Ellman, 1959).

### Total Radical-Trapping Antioxidant Potential (TRAP) Assay

Non-enzymatic total antioxidant capacity was assessed through the Total Radical-Trapping Antioxidant Potential (TRAP) assay (Lissi *et al*., 1995). This assay is based on oxidized luminol-chemiluminescence measurement induced by AAPH (2,2’-Azobis 2-amidinopropane) decomposition in glycine buffer (pH 8.6). After system (buffer + luminol + AAPH) stabilization (2h at room temperature protected from direct light), samples were added and chemiluminescence was monitored using a Wallace 1450 MicroBeta TriLux Liquid Scintillation Counter & Luminometer (Perkin Elmer). A chemiluminescence time curve was obtained and the relative 1-area under curve (1-AUC) was used for analysis (Dresch *et al.*, 2009).

### LDH assay

Lactate dehydrogenase (LDH) released by necrotic cells may be used as a marker of cell viability (Korzeniewski & Callewaert, 1983).

The release of LDH from cultured cells into the medium was quantified with a colorimetric cytotoxicity detection kit (Roche, Mannheim, Germany), used according to manufacturer instructions. In summary, culture media were collected at 48 h of culture. The samples were stored at −20ºC and measurements were performed based on the protocol provided by the manufacturer.

### Statistical Analysis

The results were expressed as the mean values ± SD calculated from three independent experiments. Except for the TRAP assays, where the non-parametric Kruskal-Wallis test was used, data were analyzed by one-way analysis of variance (ANOVA). Differences with *p*<0.05 were considered statistically significant.

## RESULTS

The analysis of the antioxidant parameters of cryopreserved ovarian tissue specimens showed that the present vitrification protocol using DMSO and EG as cryoprotectants and the metal capsule did not impair tissue antioxidant defense capacity after rewarming and after 48 h in culture. No significant tissue death was observed after 48 h, as measured by the LDH assay. In addition, the study revealed that the size of the tissue fragments did not significantly affect antioxidant defenses or viability in terms of tissue or cell death.

### GSH assay

**Figure 1:**
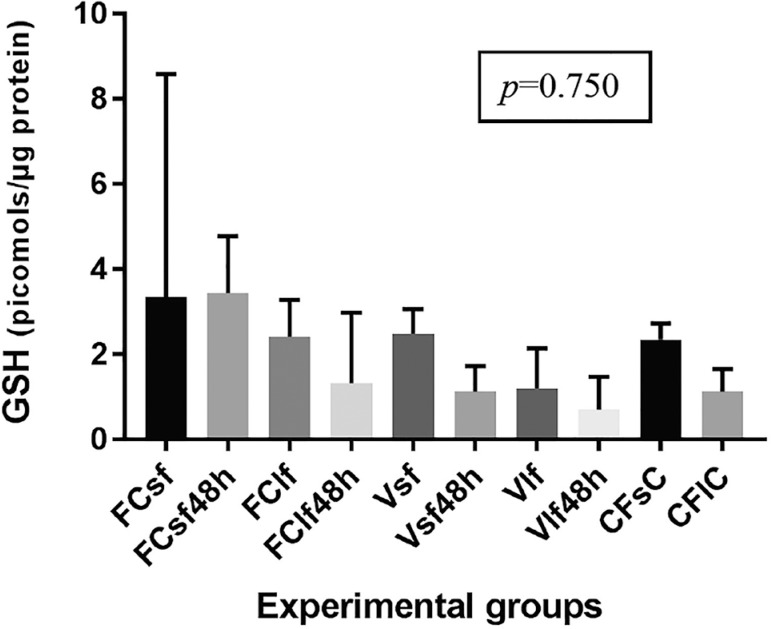
GSH values in fresh and vitrified/rewarmed ovarian tissue specimens. FCsf: Fresh control, small fragments; FCsf 48h: Fresh control, small fragments, 48 hours of culture; FClf: Fresh control, large fragments; FClf48h: Fresh control, large fragments, 48 hours of culture; Vsf: Vitrified small fragments; Vsf48h: Vitrified small fragments, 48 hours of culture; Vlf: Vitrified large fragments; Vlf48h: Vitrified large fragments, hours of culture; CfsC: Culture fresh, small control; CflC: Culture fresh, large control

The GSH levels of fresh controls (FC), vitrified specimens without culture, vitrified cultured specimens, and fresh cultured specimens of ovarian tissue are presented in Figure 1. Statistical analysis showed that there was no significant difference between fresh, vitrified and/or cultured ovarian tissue specimens for GSH content (*P*=0.750). Fresh controls presented the highest GSH levels. Fragment size did not affect GSH levels among groups. However, a more detailed examination showed that S fresh samples maintained the same GSH level after 48 hours of culture. In the vitrified groups, S and L fragments had non-significant declines in GSH levels during culture when compared to the levels observed before culture (Figure 1).

### Protein sulfhydryl assay

**Figure 2:**
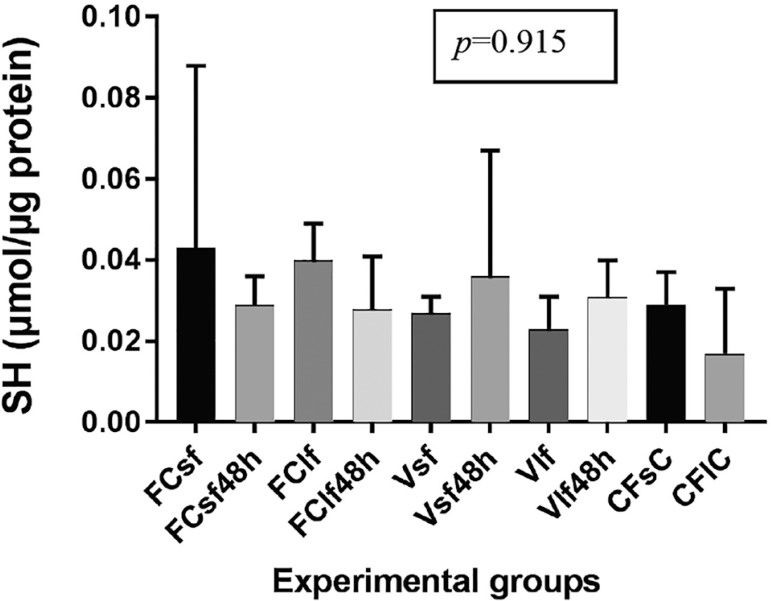
Protein sulfhydryl levels in fresh and vitrified/rewarmed ovarian tissue specimens. See Figure 1 for definitions of the control, vitrification, and culture groups

Thiol-based antioxidant levels are shown in Figure 2. The results showed no significant difference between experimental groups, regardless of fragment size (*p*=0.915). However, it is interesting to observe that protein sulfhydryl levels declined when S and L fresh samples were cultured for 48 hours. Conversely, rewarmed samples showed higher protein sulfhydryl levels and values closer to the ones seen in fresh controls, after 48 hours of culture (Figure 2).

### TRAP assay

**Figure 3:**
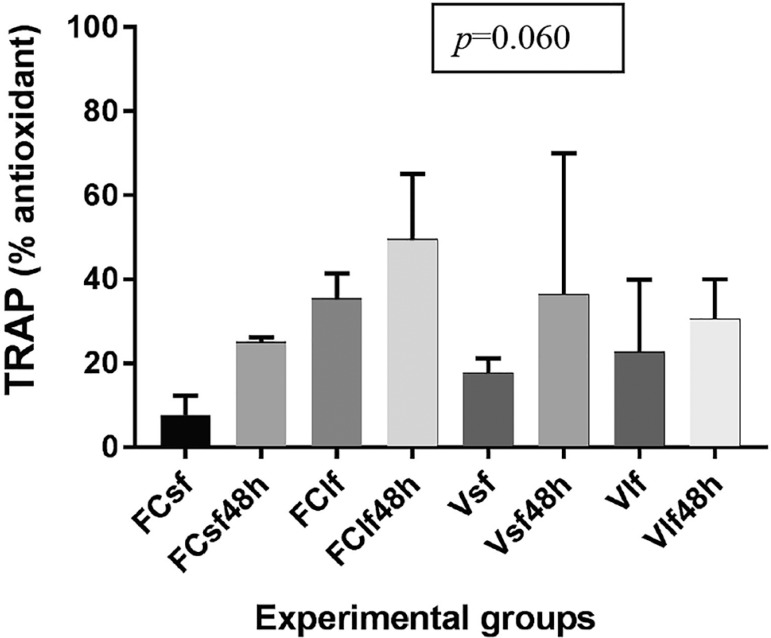
TRAP values in fresh and vitrified/rewarmed ovarian tissue specimens. See Figure 1 for definitions of the control, vitrification, and culture groups

Figure 3 shows TRAP levels for all experimental groups and controls. In the two culture control groups, small and large fragments were not included in the analysis because there were only two repeats instead of three. There were no significant differences in TRAP levels (*p*=0.060) in S, L, vitrified, vitrified and cultured, and non-vitrified controls (Figure 3).

### Detection of LDH production in cultured ovarian tissue specimens

**Figure 4:**
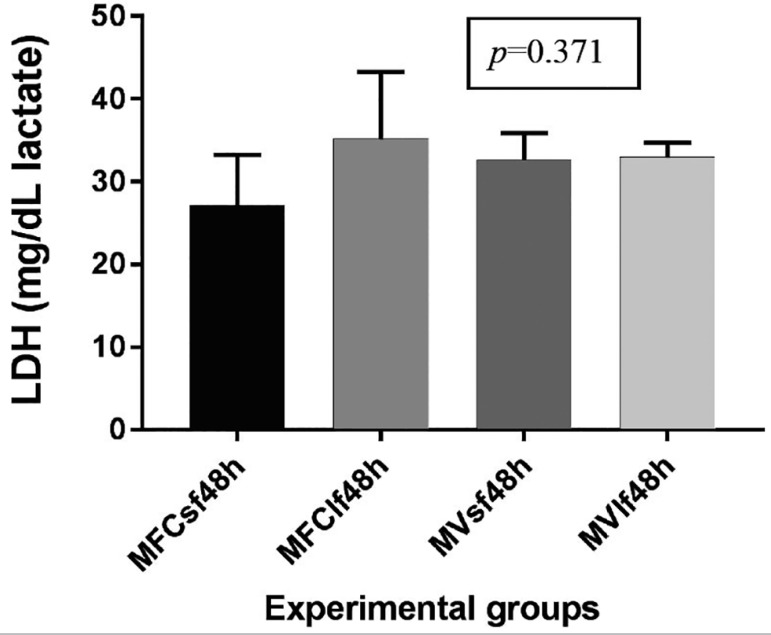
LDH measurements values in fresh and vitrified/rewarmed ovarian tissue specimens after 48 hours of culture. MFCsf48h: Medium from fresh control of small fresh fragment, cultured for 48 hours; MFClf48h: Medium from fresh control of large fresh fragment, cultured for 48 hours; MVsf48h: Medium from Vitrified small fragment, cultured for 48 hours; MVlf48h: Medium Vitrified large fragment, cultured for 48 hours

Cell viability (Preissler *et al*., 2016) was inferred from lactate dehydrogenase levels in culture media where vitrified/rewarmed, S and L fragments, and fresh samples were cultured for 48 hours. Results showed that LDH release did not differ significantly between cryopreserved and fresh, S and L fragments after 48 hours of culture (*p*=0.371) (Figure 4).

## DISCUSSION

Our results demonstrated that the protocol described in this report using a closed metal chamber for bovine ovarian tissue vitrification did not significantly alter the redox status of cryopreserved specimens. In addition, fragment size did not significantly affect post-rewarming tissue antioxidant capacity or cell death. The data showed that there was no significant alteration in GSH or sulfhydryl levels, TRAP capacity or LDH production, in S and L vitrified/rewarmed tissue specimens when compared to fresh controls immediately after rewarming and after 48 hours of culture.

Despite the abundant literature on the vitrification of isolated ovarian follicles and tissues from different species using different protocols, reported results vary in terms of cell and tissue viability or live births. More importantly, most vitrification methods use open systems to expose gametes, embryos, and tissue specimens to liquid nitrogen. This is a critical point to be taken into consideration, particularly when human tissue samples are considered. Previous reports showed that liquid nitrogen might induce microbial contamination of biological specimens (Bielanski *et al*., 2000). Thus, a safe and efficient method to vitrify human ovarian samples is urgently needed. Our group has developed a metal chamber in which bovine and zebrafish ovarian tissues were vitrified without any direct contact with LN2. Histological analysis of the rewarmed samples showed well-preserved primordial and primary follicles, in addition to stroma cells and collagen fibers (Aquino *et al.*, 2014; Marques *et al.*, 2015).

In order to perform a physiological assessment of rewarmed ovarian tissue, the biochemical protocols typically used for fluids and cell suspensions were adjusted to process bovine ovarian tissue and assess antioxidant capacity and cell death after cryopreservation and culture. The use of the bovine model is justified by the fact that it has a similar cellular and extracellular matrix composition and anatomical organization to the human ovary.

The generation of free reactive oxygen species is a normal physiological process in living cells. However, when ROS production exceeds cell defense capacity, cell DNA may be damaged. Cell damage due to free oxygen radicals produced during cryopreservation procedures is known to affect cell quality. In 1997, Mazur put forward the hypothesis that Oxyrase, an *Escherichia coli* membrane preparation (Adler, 1990) that reduces oxygen levels in freezing solutions, might protect cells by reducing the production of superoxide free radicals and other reactive oxygen species during the cryopreservation of mammalian sperm (Mazur *et al*., 2000). Thus, vitrification protocols in which there is exposure to relatively high levels of cryoprotectants, as in freezing procedures, may induce an excessive generation of ROS in cells and tissues and cause cell damage and impairment or total loss of viability after rewarming. GSH measurements made in this study showed that there was no statistically significant difference in the GSH levels of fresh and cryopreserved specimens. However, there was a perceptible, albeit non-significant, decline in the GSH levels of vitrified versus fresh samples during culture. This difference in GSH concentration between fresh and cryopreserved specimens during culture may account for the amount of GSH that was oxidized as a result of the vitrification process.

Vitrified specimens had a non-significant increase in protein sulfhydryl content during culture. A possible explanation is that vitrification/rewarming media and processes are stressing to the fragments, which prompts the oxidation of sulfhydryl groups. Culture medium is supposed to be a stable and ideal environment for cell survival; therefore, sulfhydryl groups are not oxidized and vital cell processes are preserved.

TRAP assay results were not statistically different among groups; all groups, fresh and cryopreserved, had high TRAP values after 48 hours of culture. As mentioned above, a possible explanation is that culture conditions are cell-friendly and cells do not use their non-enzymatic antioxidant defenses as much compared to when they undergo vitrification or tissues are manipulated prior to cryopreservation or culture.

LDH is a soluble cytoplasmic enzyme present in almost all cells that is released into the extracellular space when the plasma membrane is damaged (Burd & Usategui-Gomez, 1973). Thus, LDH assays may be used to determine the amount of cell death under a given condition (Smith *et al*., 2011). The linearity of the assay allows it to be used to enumerate the proportion of necrotic cells in a sample (Chan *et al*., 2013). Cell death by necrosis is usually triggered by external factors such as toxic chemicals. The high concentrations of cryoprotectants used in vitrification might produce irreversible toxic effects on cells and tissues when the procedure is not accurately performed. Considering that the vitrification protocol used in the present study does not induce irreversible cell damage, necrosis was not expected. Our data on LDH concentrations during culture of fresh and vitrified/rewarmed tissue samples showed that there was no increase in cell death due to vitrification. Furthermore, the data indicated that the obtained LDH values (25 to 34 mg/dL) were within the physiological range expected for human plasma and cerebrospinal fluid.

Studies have shown that the vitrification of immature mouse whole ovaries produced no harmful effects on the subsequent development of isolated follicles (Haidari *et al*., 2008; Mazoochi *et al*., 2009). Other authors have demonstrated that the increase in ROS levels observed after the vitrification of mouse ovary specimens was reversed during culture after rewarming (Abedelahi *et al*., 2010; Hatami *et al*., 2014). Although ours was not a murine model, our findings corroborated the results published in the cited studies and showed that ovarian tissue vitrification produced no harmful effects on subsequent tissue viability in terms of antioxidant defense capacity and cell death.

The purpose of cryopreserving ovarian tissue is not limited to fertility preservation. Ovarian tissue cryopreservation has the potential to restore endocrine and reproductive ovarian function, a possible benefit for women not immediately interested in reproduction, but who want to have their ovarian physiology restored. Thus, despite the importance of the findings described by Abedelahi *et al*. (2010) and Hatami *et al*. (2014), follicle isolation and *in vitro* maturation might not be the better options to treat prepubertal girls or young women who do not want to depend on hormone replacement therapy to secure their female hormone profile. In addition, follicle isolation is not as easily performed in human ovaries as it is in mice, because of the larger amount of fibrous extracellular matrix in humans. Therefore, tissue fragment cryopreservation seems to be a more generally acceptable procedure for fertility preservation. Safe protocols are required to ensure the feasibility of the procedure.

The overall results reported in this paper showed that tissue specimen size was not an important factor in the vitrification of ovarian specimens performed based on the protocol described in our experiments. It is generally accepted that small specimens yield better results (Ferreira *et al*., 2010). However, comparative data between small and large tissue fragments is scarce and studies on the topic refer to slow freezing, not vitrification.

The high variability observed in some of our results was probably due to random biological variability between individual animals. The study used ovaries collected in an abattoir, which means that we had no access to important data such as age or breed of the animals, and whether they were fertile females. This situation is very similar to what may happen in a human ovarian tissue banking service, where women of different ages and backgrounds may present diseases that may or not affect their fertility and the viability of their ovarian tissue specimens after cryopreservation.

In conclusion, our data showed that vitrification of ovarian tissue fragments did not induce significant alterations in tissue antioxidant defense capacity. This is a reassuring finding, since the capability of a cell, tissue or whole organ to combat the oxidative stress induced by cryopreservation is a key factor for the survival and normal physiological function of the graft after rewarming and re-transplantation. We believe that the methodology described in this report can be safely extrapolated to human ovarian tissue banking for fertility preservation purposes.
